# Correction to “Artificial Intelligence and Automation in Evidence Synthesis: An Investigation of Methods Employed in Cochrane, Campbell Collaboration, and Environmental Evidence Reviews,” “Creating Interactive Data Dashboards for Evidence Syntheses,” and “Using GPT‐4 for Title and Abstract Screening in a Literature Review of Public Policies: A Feasibility Study”

**DOI:** 10.1002/cesm.70084

**Published:** 2026-05-16

1

K. L. Scotti, S. Young, M. A. Gainey, H. Lan, “Artificial Intelligence and Automation in Evidence Synthesis: An Investigation of Methods Employed in Cochrane, Campbell Collaboration, and Environmental Evidence Reviews.” Cochrane Evidence Synthesis and Methods 3 (2025): 1‐30, https://doi.org/10.1002/cesm.70046.

Perdue, L. A., Trevino, S. D., Grant, S., Lin, J. S. and Tanner‐Smith, E. E. (2025), “Creating Interactive Data Dashboards for Evidence Syntheses.” Cochrane Evidence Synthesis and Methods 3: e70035, https://doi.org/10.1002/cesm.70035.

Rubinstein, M., Grant, S., Griffin, B. A., Pessar, S. C. and Stein, B. D. (2025), “Using GPT‐4 for Title and Abstract Screening in a Literature Review of Public Policies: A Feasibility Study.” Cochrane Evidence Synthesis and Methods 3: e70031, https://doi.org/10.1002/cesm.70031.

In the originally published Version of Record of the above articles, the “Conflicts of Interest” statement was missing from the article text.

The following “Conflicts of Interest” section has now been inserted into the article to ensure the record is complete and transparent:

## Conflicts of Interest

The authors declare no conflicts of interest.

We apologize for this error.

